# First record of *Stigmatomyces* (Ascomycota: Laboulbeniales) on Drosophilidae from Japan

**DOI:** 10.1080/19336934.2023.2234265

**Published:** 2023-07-20

**Authors:** Izumi Yamazaki, Moe Onuma, Haruka Omiya, Tomohiko Ri, Natsumi Kanzaki, Yousuke Degawa, Kyoichi Sawamura

**Affiliations:** a Degree Programs in Life and Earth Sciences, Graduate School of Science and Technology, University of Tsukuba, Tsukuba, Japan; b Sugadaira Research Station, Mountain Science Center, University of Tsukuba, Ueda, Japan; c Kansai Research Center, Forestry and Forest Products Research Institute, Kyoto, Japan; d Faculty of Life and Environmental Sciences, University of Tsukuba, Tsukuba, Japan

**Keywords:** *Drosophila suzukii*, new host, *Scaptodrosophila*, *Scaptomyza*, spotted wing drosophila

## Abstract

Three *Stigmatomyces* species were detected on five drosophilid species from Japan. We report *Stigmatomyces majewskii* on *Drosophila rufa* and *Drosophila suzukii*, *Stigmatomyces scaptodrosophilae* on *Scaptodrosophila coracina* and *Scaptodrosophila subtilis*, and *Stigmatomyces sacaptomyzae* on *Scaptomyza graminum*. Except for *Scaptomyza graminum*, each of these species is a newly identified *Stigmatomyces* host. Our discovery that *D. suzukii* is a host of *S. majewskii* may provide new pest management approaches for this global agricultural pest insect.

## Introduction

Laboulbeniales is an order of small fungi found only on the cuticles of living arthropods. Their complete nutritional dependence from their host (obligate parasitism) differentiates them from the typical Ascomycota [1,2]. The genus *Stigmatomyces* (family Laboulbeniaceae) consists of ~200 species that are primarily found on Diptera and less frequently on Coleoptera (Staphylinidae, Coccinellidae, and Elateridae) and Hemiptera (Anthocoridae), therefore called ‘fly laboulbeniomycetes’. Though 10 *Stigmatomyces* species hosted by Drosophilidae have been described (Table 1), none have been reported from Japan. One of the authors (MO) discovered a *Stigmatomyces* species on *Drosophila suzukii* (Matsumura, 1931) in 2018, and we report on *Stigmatomyces* collections carried out in Japan between 2019 and 2022.

**Table 1.** *Stigmatomyces* species are known to be hosted by Drosophilidae.

Species	Reported in the present report
*S. entomophilus* (Peck) Thax., 1900	
*S. scaptomyzae* Thax., 1900	Yes
*S. leucophengae* Thax., 1917	
*S. varians* Thax., 1918	
*S. subinflatus* Thax., 1931	
*S. tetrandrus* Thax., 1931	
*S. variatus* Thax., 1931	
*S. majewskii* H.L.Dainat, Manier & Balazuc, 1974	Yes
*S. macanus* W. Rossi & A. Weir, 2011	
*S. scaptodrosophilae* W. Rossi & E. Christian, 2020	Yes

Our discovery of a frequent *Stigmatomyces* infection on wild Japanese *D. suzukii* has an impact on agricultural pest management. *D. suzukii* is endemic to East and Southeast Asia, but began to rapidly expand globally in the late 2000s [3–5]. Females have large serrated ovipositors (hypogynium) that cause extensive damage to ripening fruits and, as a result, is considered a costly invasive agricultural pest [3–5]. Research into management strategies for *D. suzukii* is ongoing [6] and includes investigating the use of pathogenic fungi [7]. Our discovery of a potential natural enemy of *D. suzukii* potentially opens new management strategies.

## Materials and methods

### *Determination of* Stigmatomyces *on Drosophilidae*

We collected Drosophilidae from nine locations in Japan (Fig. S1) between 2019 and 2022, with most samples coming from Fushimi (Kyoto), Sugadaira (Nagano), and Tsukuba (Ibaraki). Flies were caught primarily using bait traps containing banana and yeast. We also collected by sweeping nets over fallen fruits. Flies were anaesthetized with triethylamine and preserved in 70% ethanol before being sorted under a binocular stereo microscope (SMZ-U, Nikon, Tokyo, Japan). The different parts of individual flies were examined to determine whether *Stigmatomyces* is infected, and the infection frequency on each body part was recorded. To identify the *Stigmatomyces* species, thalli were removed from the flies using forceps and fixed in 99.9% lactate. They were mounted on glass slides and examined under a light microscope (CX23, Olympus, Tokyo, Japan) or a phase-contrast microscope (BH-2, Olympus). Images were acquired by using a single-lens reflex camera (EOS60D, Canon, Tokyo, Japan) connected with a dedicated adaptor (NY1S, Canon). The sizes of specimens were measured by using 11φ24 and NOB1 micrometres (Fuji Kogaku, Kumamoto, Japan). Scale bar was inserted to the images by using ImageJ 1.53k (https://imagej.nih.gov/ij/). We used R 4.2.1 (https://www.r-project.org) for the statistical tests.

### *Observation of grooming behaviour of* D. suzukii

The behavioural observations used an iso-female strain of *D. suzukii* (SGD001) that was collected in the Sugadaira highlands (Ueda, Nagano Prefecture) in 2017 and maintained at 25°C under a light/dark cycle (L:D = 14 h:10 h). Virgin flies used for the observations were collected without anaesthesia within 8 h of emergence and kept in male- or female-only groups prior to use. They were individually aspirated to a Petri dish (diameter, 3.6 cm; height, 1.0 cm) and observed at room temperature for 10 min under the binocular stereo microscope. Four age groups were used (4, 8, 12, and 16 days after emergence), each replicated 10 times (*n* = 40 for each sex). Because there was no difference in grooming behaviour among the age groups of the same sex, the data were pooled. *S. majewskii*-infected *D. suzukii* males were collected from the wild and maintained at 18°C under constant light before use and examined under the same conditions as above (*n* = 17).

## Results and discussion

### Stigmatomyces majewskii *H. L. Dainat, Manier & Balazuc, 1974*

**Figure 1.**
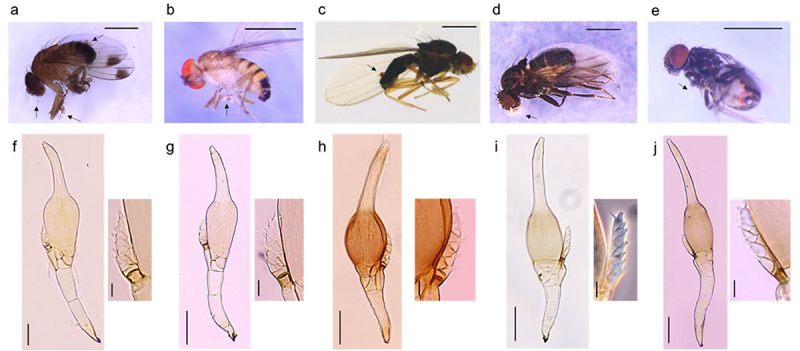
Host drosophilid species (a-e) and parasitic *Stigmatomyces* species (f-j). (a) *D. suzukii*, (b) *D. rufa*, (c) *Scaptomyza graminum*, (d) *Scaptodrosophila subtilis*, (e) *Scaptodrosophila coracina*, (f) *S. majewskii* on *D. suzukii*, (g) *S. majewskii* on *D. rufa*, (h) *S. scaptomyzae* on *Scaptomyza graminum*, (i) *S. scaptodrosophilae* on *Scaptodrosophila subtilis*, and (j) *S. scaptodrosophilae* on *Scaptodrosophila coracina*. Arrows indicate the *Stigmatomyces* infection on flies in a-e. The appendage is enlarged in inserts of f-j. The phase contrast view is shown in i and j (insert). Scales: 1 mm in a-e, 50 μm in f-j, 10 μm in the inserts.

We collected *D. suzukii* flies infected with *Stigmatomyces* from eight localities of Japan (Figures. 1(a) and S1). A *D. rufa* Kikkawa & Peng, 1938 male that was collected from Kyoto on 22 October 2022 was also found to be infected with the same *Stigmatomyces* species on tibia of his left foreleg (Figure 1(b)). Based on their diagnostic characters [8–12], they were identified as *Stigmatomyces majewskii* (Figure 1(f),(g)). *S*. *majewskii* has a perithecial neck as long as the venter, transparent appendages, and an appendage axis composed of four cells (differentiating it from the axis of *Stigmatomyces entomophilus* (Peck) Thax., 1900 that is composed of six cells). See Appendix A for the difference between *S*. *majewskii* and its close relatives. The length of thalli was 410–590 μm (*n* = 13).

Although the number of our primary collection sites was limited, our emphasis was placed on identifying infected *D. suzukii* at every locality (Fig. S1). The collection at Fukuoka (24–29 September 2021) is an exception: no infected *D. suzukii* were recorded from 26 females and 199 males. Interestingly, our *D. suzukii* collection (September 2019-October 2022) always exhibited a biased sex ratio (χ^2^ = 976.31, d.f. = 1, *p* < 2.2E–16), with 89.0% (*n* = 2734) being males. Furthermore, the frequency of infection was significantly higher (χ^2^ = 35.495, d.f. = 1, *p* = 2.557E–9) in males (16.5%, *n* = 2432) than females (3.3%, *n* = 302). We observed the frequency of infection to fluctuate seasonally (Appendix B). *D. suzukii* displayed two seasonal morphs, a summer morph with a lighter body colour and a winter morph with a darker body colour [13]. Both morphs were found infected with *S. majewskii* (Appendix B).

Why did our collection include more *D. suzukii* males (frequently *Stigmatomyses*-infected males) than females? We propose five hypotheses for this observation: 1) Our collection method (primarily banana-yeast bait traps) may have been more attractive to males (especially for active, infected males) than females; females may prefer odours from ripening fruits. 2) Males may have the tendency to get infected more easily than females due to unknown sexually dimorphic immune factors or due to higher male–male transmission resulting from higher male-to-male contact compared to female–female contact. 3) There could be an increased nutritional content that the males provide to *S. majewskii* compared to females. 4) Females may have a higher efficiency in removing *S. majewskii* compared to males. 5) Infected females may have lower fitness in the field and thus are less numerous. To determine which hypothesis is correct, we are analysing the mechanisms of *S. majewskii* infection in *D. suzukii*. Further analyses will be reported in a separate paper.

**Figure 2.**
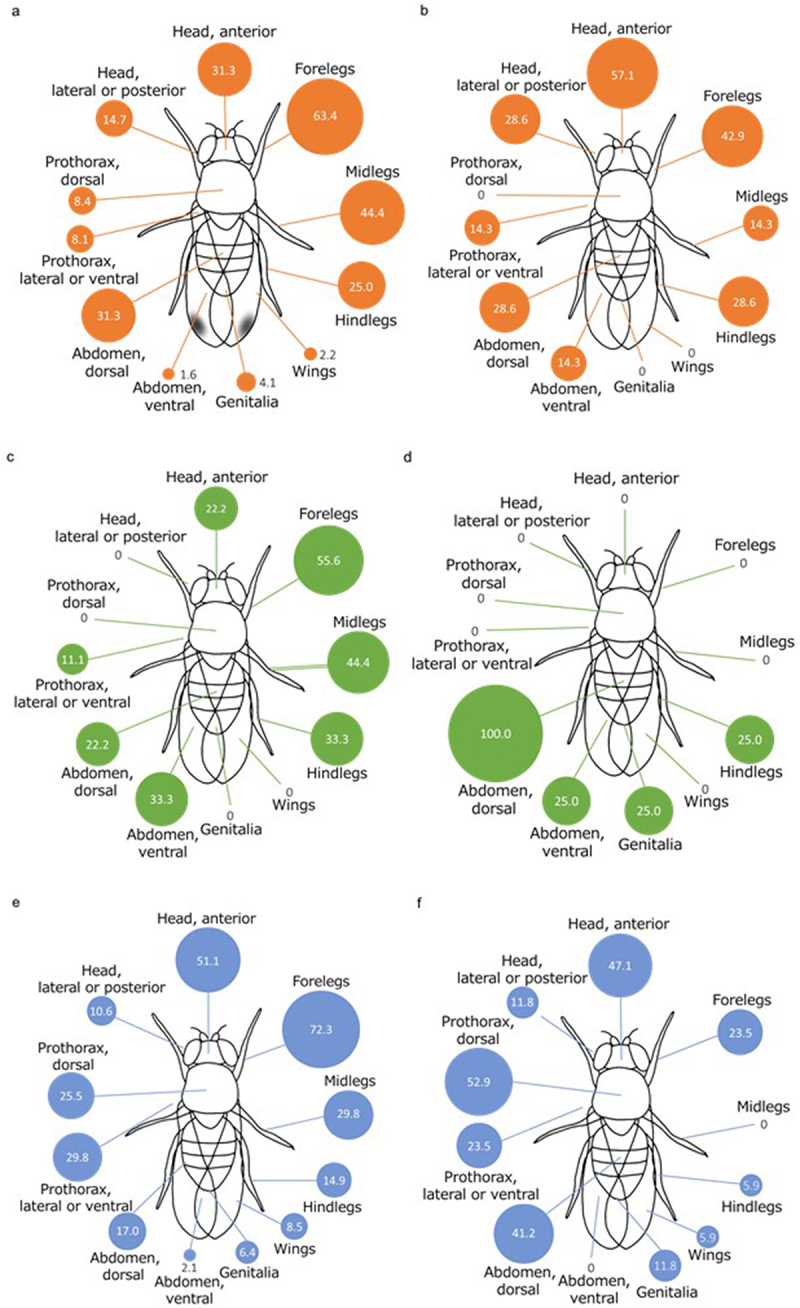
Frequency of *Stigmatomyces* infection on body parts: (a) *S. majewskii* on *D. suzukii* males (*n* = 320), (b) *S. majewskii* on *D. suzukii* females (*n* = 7), (c) *S. scaptomyzae* on *Scaptomyza graminum* males (*n* = 9), (d) *S. scaptomyzae* on *Scaptomyza graminum* females (*n* = 4), (e) *S. scaptodrosophilae* on *Scaptodrosophila subtilis* males (*n* = 47), and (f) *S. scaptodrosophilae* on *Scaptodrosophila subtilis* females (*n* = 17).

The *S. majewskii* infection on *D. suzukii* males (*n* = 320) was frequently observed on their forelegs (63.4%; especially on femur (50.0%), tibia (24.7%)), midlegs (44.4%), anterior head (31.3%; such as, maxillary palp, frons, labella, antenna, arista and edge of the compound eyes), and dorsal abdomen (31.3%) (Figures. 2(a) and S2(a)). On *D. suzukii* females (*n* = 7), the infections tend to be located on the anterior of their heads (57.1%), forelegs (42.9%), hindlegs (28.6%), lateral or posterior head (28.6%), and dorsal abdomen (28.6%) (Figures. 2(b) and S2(b)). In both sexes, the infection was seen on the marginal and proximal regions of the wings and on the left or right positions of the dorsal abdomen (rarely on the mid position). The infection was not observed in the compound eyes, although immature thalli were detected. *S. majewskii* can infect the entire body of *D. suzukii* but are probably removed by using their legs (Appendix C). This differs from guava fruit flies (*Anasterepha striata* Schiner, 1868), where the *Stigmatomyces* (*S*. *aciurae* Thaxt., 1917 and *S. verruculosus* Thaxt., 1917) infection is restricted to the areas of sexual contact [14]. We arranged our observations of *S. majewskii* according to their developmental stage (Appendix D). Interestingly, we collected preliminary data that suggests that *S. majewskii* may include dioecious individuals (Appendix E).

### Stigmatomyces scaptomyzae *Thaxt., 1901*

*Stigmatomyces scaptomyzae* is common in Europe, Africa, and North and South America, and observed on *Scaptomyza pallida* (Zetterstedt, 1847) and *Scaptomyza graminum* (Fallén, 1823) [9,15,16]. Based on its unique molecular synapomorphies, it has been suggested that this species be moved to the genus *Appendiculina* [17]. One of the host species, *Scaptomyza graminum*, can be collected in late autumn by net sweeping over grasslands and gardens because Caryophyllaceae is the primary food source of larva [18]. We collected *Stigmatomyces*-infected *Scaptomyza graminum* from Hachioji, Tokyo on 12 November 2022 and from Tsukuba, Ibaraki on 14–17 November 2022 (Figure 1(c),(h)). The infection frequency was 7.8% in females (*n* = 51) and 14.3% in males (*n* = 49). No infections were detected on *Scaptomyza pallia* females (*n* = 18) or males (*n* = 40) collected simultaneously at Tsukuba.

We identified the species to be *S. scaptomyzae* based on its diagnostic characters: the axis of the appendage is composed of five cells, the stalk-cell of the appendage is broader than the brown and orthogonal basal cell, and the perithecium is brown. The length of thalli was 220–260 μm (*n* = 5). On *Scaptomyza graminum* males (*n* = 9), the infection was frequently seen on legs and abdomen, especially at the ventral side (Figures. 2(c) and S2(c)). On the females (*n* = 4), the infection was restricted to hindlegs, genitalia, and abdomen, especially at the dorsal side (Figures. 2(d) and S2(d)). Therefore, we suspect that the infection was partly caused by intersexual contact. Furthermore, we have preliminary data suggesting that *S. scaptomyzae* may include dioecious individuals (Appendix E).

### Stigmatomyces scaptodrosophilae *W. Rossi & E. Christian, 2020*

*Stigmatomyces scaptodrosophilae* was recently described based on samples found on *Scaptodrosophila deflexa* (Duda, 1924) from Ukraine and Austria and a third from Kenya on *Scaptodrosophila* sp [19]. We discovered this species in Fushimi, Kyoto on *Scaptodrosophila coracina* (Kikkawa & Peng, 1938) and *Scaptodrosophila subtilis* (Kikkawa & Peng, 1938) and in the Sugadaira highlands, Nagano on *Scaptodrosophila coracina* (Figure 1(d),(e),(i),(j)). We verified that the axis of the appendage is composed of five to six cells, the stalk-cell of the appendage is broader than the orthogonal basal cell, and the stalk-cell of the perithecium is shorter on the inner side than the stalk-cell of the appendage while being separated from the secondary stalk-cell by an obliquely hollow septum. The length of thalli on *Scaptodrosophila coracina* was 410–450 μm (*n* = 3) and was 310–330 μm on *Scaptodrosophila subtilis* (*n* = 4). The cause of the different thalli sizes remains unclear but may be related to genetic differences of *S. scaptodrosophilae* or the nutritional differences provided by the two hosts. Based on our samples from Kyoto (6 April - 21 June 2022) and Sugadaira (12 September - 11 October 2022), the frequency of infection was 4.6% on *Scaptodrosophila subtilis* females (*n* = 432) and 15.9% on males (*n* = 302). The frequency of infection on *Scaptodrosophila coracina* females (*n* = 773) and males (*n* = 3042) was 0.3% in both sexes.

Our observations of *S. scaptodrosophilae* on *Scaptodrosophila subtilis* males (*n* = 47) revealed that the forelegs were the most common site of infection (72.3%; especially on femur (59.6%) and on coxa (23.4%)), followed by the anterior head (51.1%), midlegs (29.8%), and ventral or lateral thorax (29.8%) (Figures. 2(e) and S2(e)). On *Scaptodrosophila subtilis* females (*n* = 17), the infection was seen on dorsal thorax (52.9%), anterior head (47.1%) and dorsal abdomen (41.2%), with low infection frequency on the midlegs (0%) and hindlegs (5.9%) (Figures. 2(f) and S2(f)). The positions of the infection on *Scaptodrosophila coracina* males (*n* = 10) and females (*n* = 2) have been indicated in Figure S3. Furthermore, we have preliminary data suggesting that *S. scaptodrosophilae* may include dioecious individuals (Appendix E).

## Concluding remarks

In the present report, we identified three *Stigmatomyces* species on five Japanese drosophilid species. Although the three *Stigmatomyces* species had been described in other countries, most of their hosts reported here (four species) are new discoveries. Because investigations into the natural history of *Stigmatomyces* in Asia have so far been limited [20], we suspect that more *Stigmatomyces* species and their hosts remain to be identified. Additional studies like this one will also help us to understand the impact that *Stigmatomyces* has on the population dynamics of Drosophilidae in nature. Of particular importance are the effects of *S. majewskii* on the agricultural pest *D. suzukii*. As *D. suzukii* is a global invasive species that originated in Asia, it may be free of natural enemies within in its new habitats. The introduction of predators, parasites, parasitoids, and pathogens presents attractive management strategies, and *S. majewskii* provides a new and potentially important option to investigate further.

## Supplementary Material

Supplemental Material

## Data Availability

The authors confirm that the data supporting the findings of this study are available within the article [and/or] its supplementary materials.

## Appendix A The difference between *S. majewskii* and its close relatives

*S*. *majewskii* has previously been recorded in various localities outside of Japan [10,11,16,21,22]. In Europe, *S. majewskii* has been found on various species of the *Drosophila obscura* subgroup (*D. obscura* Fallén, 1823, *D. subobscura* Collin, 1936, and *D. ambigua* Pomini, 1940), as well as on *Scaptodrosophila rufifrons* (Loew, 1873) (referred to as (≡) *D. rufifrons* in previous papers). In Zimbabwe, *S. majewskii* has been reported on a species belonging the *D. montium* group and in Taiwan on *D*. cf. *takahashii* Sturtevant, 1927. A close relative to *S. majewskii* is *S. entomophilus* (Peck) Thax., 1900 (synonymized to as (=) *S. drosophilae* Thax., 1931). *S. entomophilus* is distributed in North and South America and Europe [8,9,23,24]. Known hosts of *S. entomophilus* are *D. funebris* (Fabricius, 1787), a species of the *D. repleta* group, and *Hirtodrosophila confusa* (Staeger, 1844) (≡ *D. confusa*) [8,9,24]. *S. entomophilus* (≡ *Appendicularia entomophilus*) was originally described on *D. nigricornis* from New York [25]; however, this *Drosophila* is a *nomina nuda* [26]. Interestingly, hosts of these two *Stigmatomyces* species differ in their taxonomic position under the genus *Drosophila*: *S. entomophilus* is seen on species within the subgenus *Drosophila*, while *S. majewskii* is seen on species within the subgenus *Sophophora* [11,12]. Though not identified (W. Starmer, personal communication), the *Stigmatomyces* sp. was reported from New York on species from the *D. affinis* subgroup (*D. affinis* Sturtevant, 1916, *D. algonquin* Sturtevant & Dobzhansky, 1936, and *D. athabasca* Sturtevant & Dobzhansky, 1936) [27]. Moreover, the fact that the *D. affinis* subgroup is a sister subgroup of the *D. obscura* subgroup [28] provides additional intrigue to the relationship between *S. majewskii* and *Stigmatomyces* sp. of reference [27].

## Appendix B Seasonal fluctuation of *D. suzukii* and the *S. majewskii* infection

Here, we briefly describe the seasonal fluctuation in the host population and the infection frequency based on the data collected at three localities. The *D. suzukii* population grows in spring/autumn in Kyoto and Tsukuba (*D. suzukii* can be collected throughout the year, except during hot summers), while it presumably cannot survive the cold winters in the Sugadaira highlands. The infection frequency was high in the season of *D. suzukii* population growth, with a time lag for the increase. The infection frequency in male *D. suzukii* was high during April–June and September–November in Kyoto (maximum 29.8%, *n* = 47 in May 2022), during March–April and October–November in Tsukuba (maximum 25.0%, *n* = 48 in November 2021), and during September in Sugadaira (maximum 31.2%, *n* = 456 in September 2021).

*D*. *suzukii* has a summer and a winter morph. As indicated in Figure S4, at Kyoto, the winter morph appears as early as November and both the morphs overwinter, although winter morphs constitute majority of the survivors. The frequency of the male summer morph increased in spring (March, 12.2%, *n* = 41; April, 53.1%, *n* = 98; May, 53.2%, *n* = 47; June, 89.2%, *n* = 37). The infection frequency was significantly higher (χ^2^ = 9.1077, d.f. = 1, *p* = 0.002545) in the summer morph males (28.4%, *n* = 148) than the winter morph males (13.3%, *n* = 143). Interestingly, the infection frequency of the winter morph males increased in spring (March, 5.6%, *n* = 36; April, 13.0%, *n* = 46; May, 31.8%, *n* = 22; June, 75.0%, *n* = 4). This increase in infection in the male winter morphs may be attributed to their higher susceptibility to infections during this season. Otherwise, these males would have survived longer.

## Appendix C Grooming behaviour of *D. suzukii*

Grooming is a typical fly behaviour in which their legs are used to remove foreign substances from their body ([29] for *D. melanogaster*). We observed individual unmated *D. suzukii* males and females groom over 10-minute intervals (*n* = 40 for each sex) (Fig. S5). We observed the forelegs to be used for grooming the head most frequently, while it was used the least frequently for the prothorax. The hindlegs were observed to groom the prothorax, wings, and the abdomen. Tarsi were rubbed against each other using two or three legs, while the femur and tibia were groomed using tarsus of the other legs. The grooming rates of the head, wings, and tarsi were high, but that of the genitalia, femur and tibia were low (Fig. S6). The legs seldom reached the dorsal abdomen nor the femur and tibia of the midlegs.

We observed grooming of individual wild *D. suzukii* males infected with *S. majewskii* (*n* = 17). All males exhibited grooming during the 10 min observation period, and 12/17 males were observed to touch the infected area. Examples of the contact with *S. majewskii* are listed in Table S1. Though the legs never touched the base of the thalli or removed *S. majewskii*, the possibility of removal at earlier stages of *S. majewskii* development (including spores) cannot be ruled out. The grooming behaviour might affect the *S. majewskii* infection site on the *D. suzukii* body (Figures. 2(a), (b), S2(a), (b)). One possibility is the shake-off of *S. majewskii*, for example, from the wings. Grooming may also propagate the *S. majewskii* infection to other body parts of *D. suzukii*, as has been suggested in the infection of *Stigmatomyces ceratophorus* Whisler, 1968 on *Fannia canicularis* Linnaeus, 1761 (lesser house fly) [30].

## Appendix D Staging of *S. majewskii* development

While observing *S. majewskii* infections, a range of developmental stages were observed. Spindle-shaped, transparent objects, which are presumably ascospores, were seen around matured thalli (Fig. S7(a)). The smallest individuals (35 μm) on the *D. suzukii* cuticle were the two-cell stage (Fig. S7(b)), which were sometimes truncated in the densely infected area (Fig. S7(b’)). Under the microscope, these areas appear as scattered black dots due to dark pigmentation at the base of the thalli. This is presumably the result of grooming. Figure S7(c) is the stage in which the ascogonium and appendage have similar length. Figure S7(d) represents the stage in which the neck is growing but the perithecium has not been swollen. Finally, Figure S7(e) is the stage in which the morphology is similar to the mature thalli, but still smaller.

## Appendix E Potential dioecious individuals of *Stigmatomyces*

Most of the Laboulbeniales are thought to be monoecious, but dioecious species have also been described [31,32]. Interestingly, our slide observation of *S. majewskii* (*n* = 26), *S. scaptomyzae* (*n* = 12), and *S. scaptodrosophilae* (*n* = 13) suggests the presence of dioecious individuals in the three *Stigmatomyces* species. One *S. majewskii*, presumably a “male”, had an appendage but not the perithecium (Fig. S8(a)). In contrast, one *S. scaptomyzae* and one *S. scaptodrosophilae*, presumably “females”, had the perithecium but not the appendage (Fig. S8(b), (c)). We also observed one *S. majewskii*, one *S. scaptomyzae*, and one *S. scaptodrosophilae* in which the perithecium and the neck were long (and had ascospores) but the appendage was immature (or had a smaller number of cells in the appendage), suggesting non-self-fertilization (Fig. S8(d)-(f)).

## References

[1] Benjamin RK. Laboulbeniomycetes. In: Ainsworth G, Sparrow F Sussman A, editors The fungi – an advanced treatise. Vol. 4a. New York: Academic Press; 1973. pp. 223–9.

[2] Tavares II. Laboulbeniales (fungi, ascomycetes). Mycologia memoir 9. The New York Botanical Garden. 1985.

[3] Asplen MK, Anfora G, Biondi A, et al. Invasion biology of spotted wing drosophila (*drosophila suzukii*): a global perspective and future priorities. J Pest Sci. 2015;88(3):469–494. doi: 10.1007/s10340-015-0681-z

[4] Walsh DB, Bolda MP, Goodhue RE, et al. *Drosophila suzukii* (Diptera: Drosophilidae): invasive pest of ripening soft fruit expanding its geographic range and damage potential. J Integr Pest Manage. 2011;2(1):G1–G7. doi: 10.1603/IPM10010

[5] Garcia FRM, Lasa R, Funes CF, et al. *Drosophila suzukii* management in Latin America: current status and perspectives. J Econ Entomol. 2022;115(4):1008–1023. doi: 10.1093/jee/toac052 35595171

[6] Tait G, Mermer S, Stockton D, et al. *Drosophila suzukii* (Diptera: Drosophilidae): a decade of research towards a sustainable integrated pest management program. J Econ Entomol. 2021;114(5):1950–1974. doi: 10.1093/jee/toab158 34516634

[7] Becher PG, Jensen RE, Natsopoulos ME, et al. Infection of *drosophila suzukii* with the obligate insect-pathogenic fungus *Entomophthora muscae*. J Pest Sci. 2018;91(2):781–787. doi: 10.1007/s10340-017-0915-3 PMC5847158 29568251

[8] Weir A, Rossi W. Laboulbeniales parasitic on British Diptera. Mycol Res. 1995;99(7):841–849. doi: 10.1016/S0953-7562(09)80739-9

[9] Rossi W, Máca J. Notes on the laboulbeniales (ascomycetes) from the Czech republic. Sydowia. 2006;58:110–124.

[10] Haelewaters D, van Wielink P, van Zuijlen JW, et al. New records of laboulbeniales (fungi, Ascomycota) for the Netherlands. Entom Berichten. 2012;72:175–183.

[11] Rossi W, Santamaría S, Andrade R. Notes on the laboulbeniales (Ascomycota) parasitic on Diptera from Portugal and other countries. Plant Biosys. 2013;147(3):730–742. doi: 10.1080/11263504.2012.753132

[12] Haelewaters D, De Kesel A. Checklist of thallus-forming laboulbeniomycetes from Belgium and the Netherlands, including *hesperomyces halyziae* and *laboulbenia quarantenae* spp. nov. MycoKeys. 2020;71:23–86. doi: 10.3897/mycokeys.71.53421 32831551 PMC7410850

[13] Toxopeus J, Jakobs R, Ferguson LV, et al. Reproductive arrest and stress resistance in winter-acclimated *Drosophila suzukii*. J Insect Physiol. 2016;89:37–51. doi:10.1016/j.jinsphys.2016.03.006 27039032

[14] Hedström I. *Stigmatomyces* species on guava fruit flies in seasonal and non-seasonal neotropical forest environments. Mycol Res. 1994;98(4):403–407. doi: 10.1016/S0953-7562(09)81196-9

[15] Thaxter R. Preliminary diagnoses of new species of Laboulbeniaceae. III Proc Amer Acad Arts. 1901;36(23):395–414. doi: 10.2307/20021044

[16] Christian E. The coccinellid parasite *hesperomyces virescens* and further species of the order Laboulbeniales (Ascomycotina) new to Austria. Ann Naturhist Mus Wien. 2001;103B:599–603.

[17] Haeleswaters D, Dima B, Abdel-Hafiz AII, et al. Fungal systematics and evolution: FUSE 6. Sydowia. 2020;72:231–356.

[18] Hackman W. On the genus *Scaptomyza* Hardy (Dipt., Drosophilidae) with descriptions of new species from various parts of the world. Acta Zool Fenn. 1959;97:1–73.

[19] Rossi W, Christian E. Laboulbeniales (Ascomycota) from Austria and neighboring areas. Sydowia. 2020;72:149–161.

[20] Rossi W, Weir A. New species of *Stigmatomyces* from Asia. Mycologia. 2011;103(1):131–134. doi: 10.3852/10-010 20943550

[21] Dainat H, Manier JF, Balazuc J. *Stigmatomyces majewskii* n. sp., *Stigmatomyces papuanus* Thaxter 1901, Laboulbénials parasites de Diptères Acalyptérés. Bull Soc Myc Fr. 1974;90:171–178.

[22] Rossi W, Máca J, Vavra J. New records of Laboulbeniales (Ascomycota) from the Czech Republic and Slovakia. Polish Bot J. 2010;55:343–351.

[23] Thaxter R. Contribution toward a monograph of the Laboulbeniaceae. Part 5. Mem Am Acad Arts Sci. 1931;16:1–435. doi: 10.2307/25058136

[24] Rossi W. New or interesting Laboulbeniales parasitic on Diptera from Bolivia. Mycologia. 1998;90(6):1047–1054. doi: 10.1080/00275514.1998.12027004

[25] Peck CH. Report of the botanist (1884). Ann Rep New York St Mus Natl Hist. 1885;38:77–138.

[26] Brake I, Bächli G. Drosophilidae (Diptera). World catalogue of insects. Vol. 9. Stenstrup, Denmark: Apollo Books; 2008.

[27] Starmer WT, Weir A. Laboulbeniales associated with the *Drosophila affinis* subgroup in central New York. Drosophila Inf Serv. 2001;84:22–24.

[28] O’Grady PM, DeSalle R. Phylogeny of the genus *Drosophila*. Genetics. 2018;209(1):1–25. doi: 10.1534/genetics.117.300583 29716983 PMC5937177

[29] Szebenyi AL. Cleaning behaviour in *Drosophila melanogaster*. Anim Behav. 1969;17(4):641–651. doi: 10.1016/S0003-3472(69)80006-0

[30] Whisler HC. Experimental studies with a new species of *Stigmatomyces* (Laboulbeniales). Mycologia. 1968;60(1):65–75. doi: 10.1080/00275514.1968.12018548 5644960

[31] Benjamin RK, Shanor L. Discovery of dioecism in *Laboulbenia formicarum*. Science. 1950;111(2872):33–34. doi: 10.1126/science.111.2872.33 15398818

[32] Benjamin RK. Laboulbeniales on semiaquatic Hemiptera. V. Triceromyces Aliso J Syst Flor Bot. 1986;11(3):245–278. doi: 10.5642/aliso.19861103.02

