# Supplementary figures and images for: First record of Stigmatomyces (Ascomycota: Laboulbeniales) on Drosophilidae from Japan
Source: Fly (Austin). 2023 Jul 20;17(1):2234265. doi: 10.1080/19336934.2023.2234265 (PMC10361133; doi:10.1080/19336934.2023.2234265)

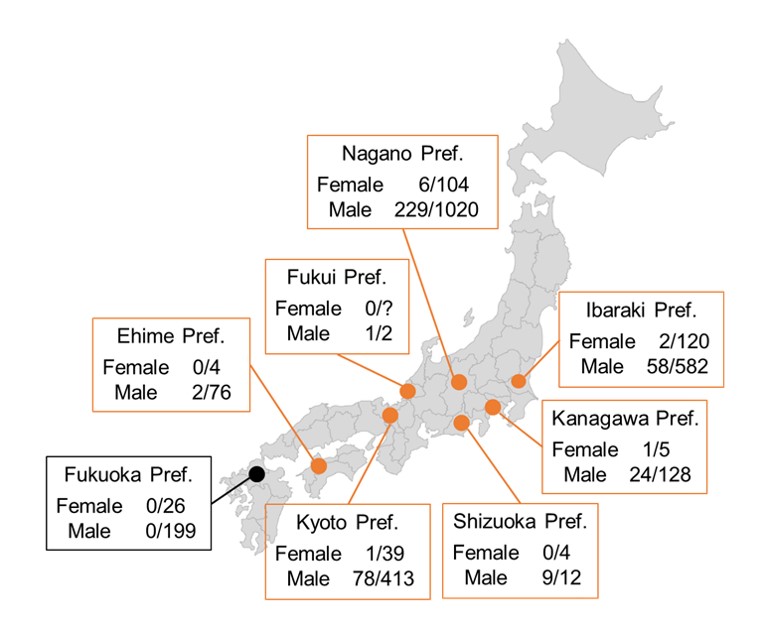

Supplement: Supplemental Material [file KFLY_A_2234265_SM3497.zip › Yamazaki et al FigS1.jpg]

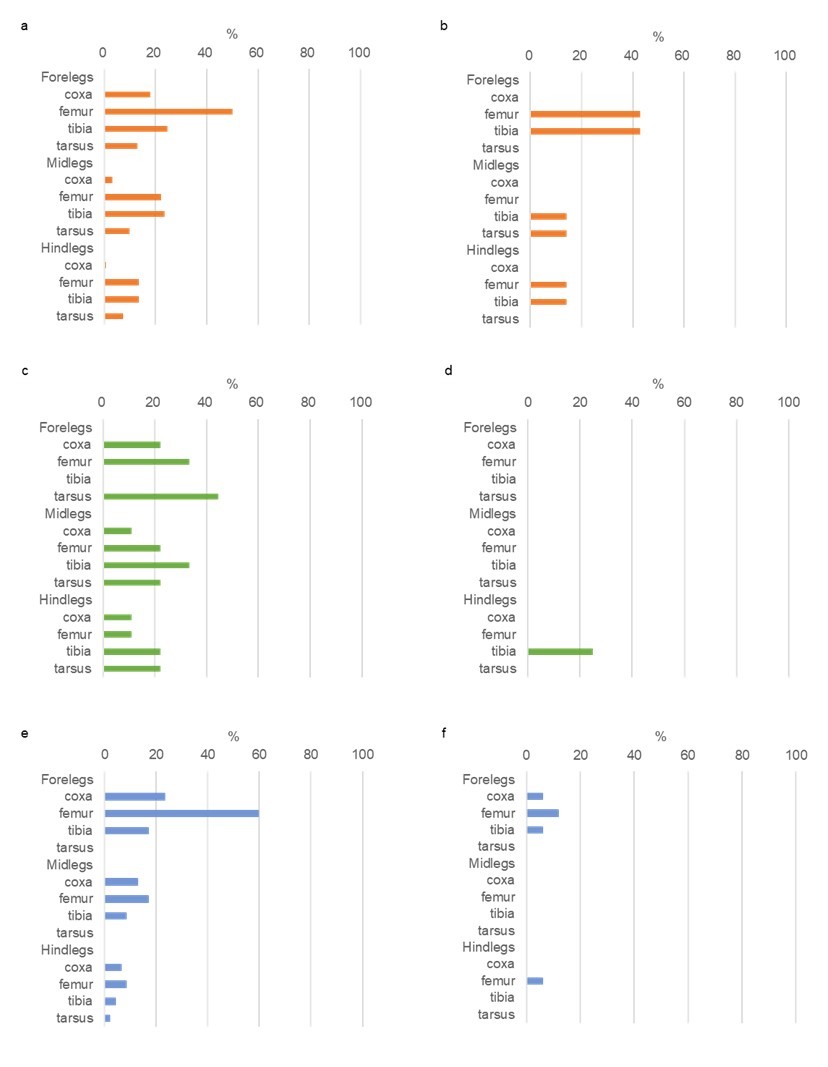

Supplement: Supplemental Material [file KFLY_A_2234265_SM3497.zip › Yamazaki et al FigS2.jpg]

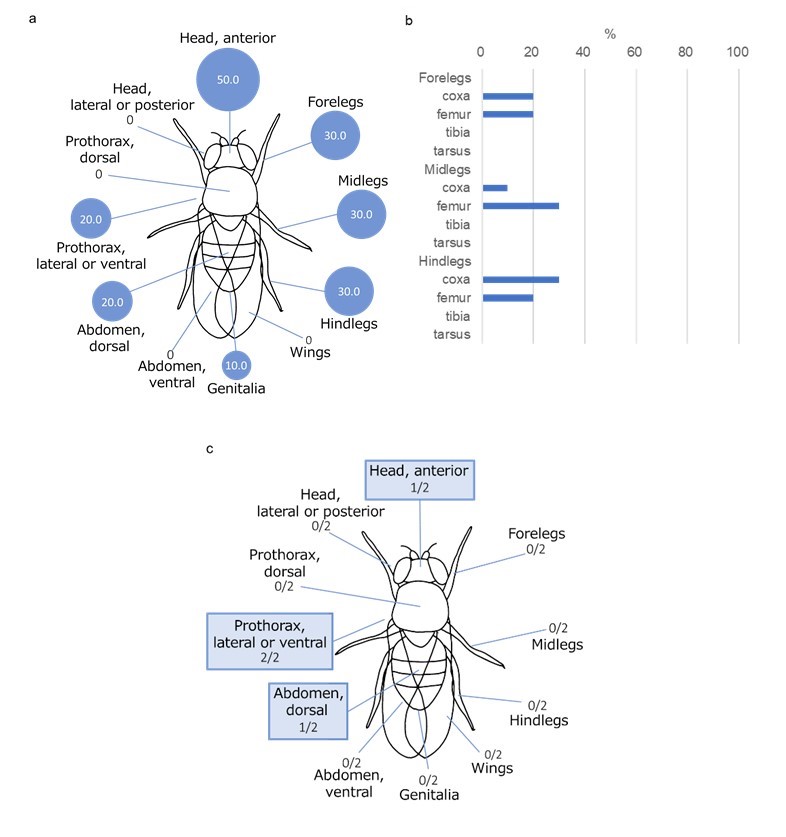

Supplement: Supplemental Material [file KFLY_A_2234265_SM3497.zip › Yamazaki et al FigS3.jpg]

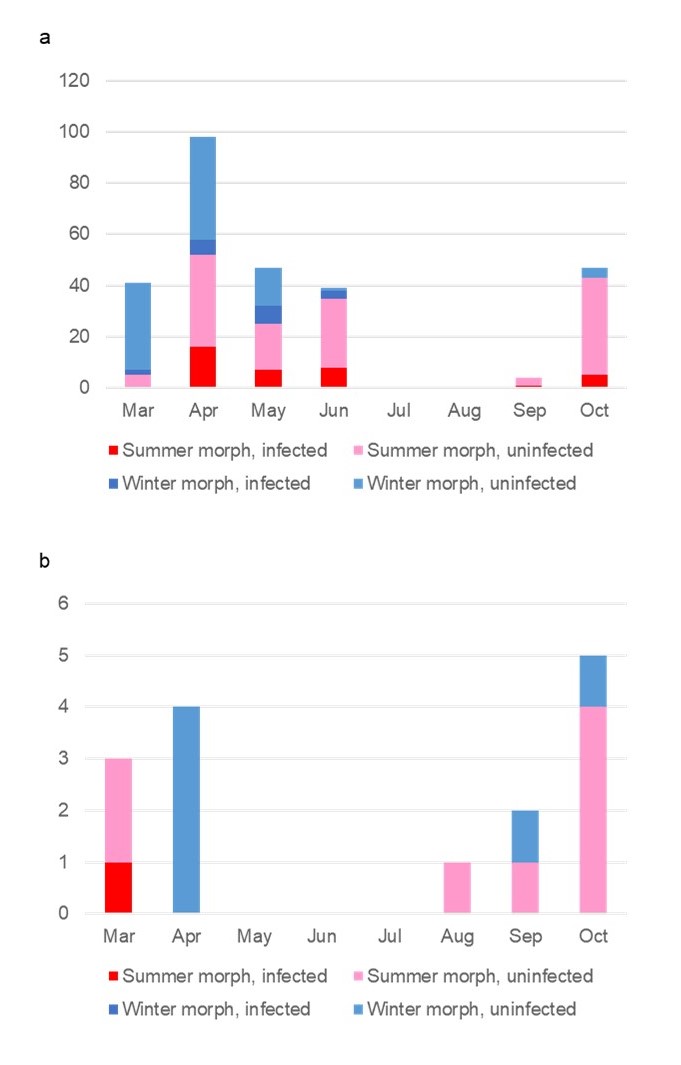

Supplement: Supplemental Material [file KFLY_A_2234265_SM3497.zip › Yamazaki et al FigS4.jpg]

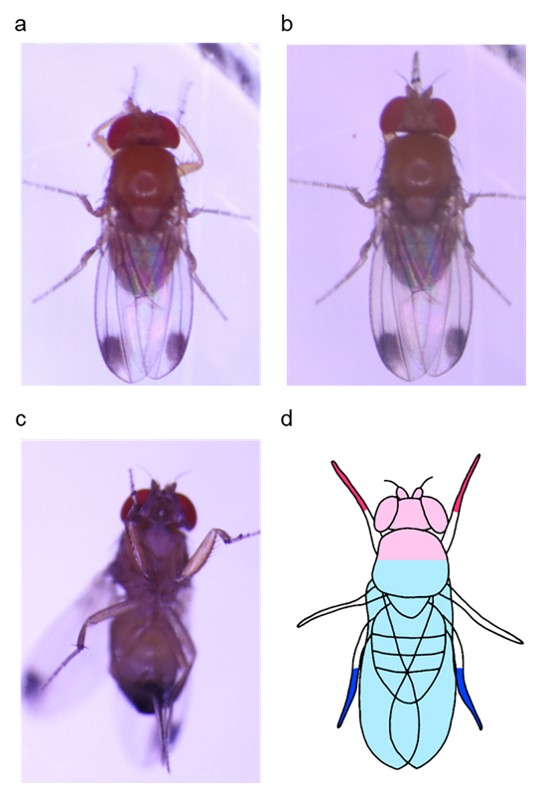

Supplement: Supplemental Material [file KFLY_A_2234265_SM3497.zip › Yamazaki et al FigS5.jpg]

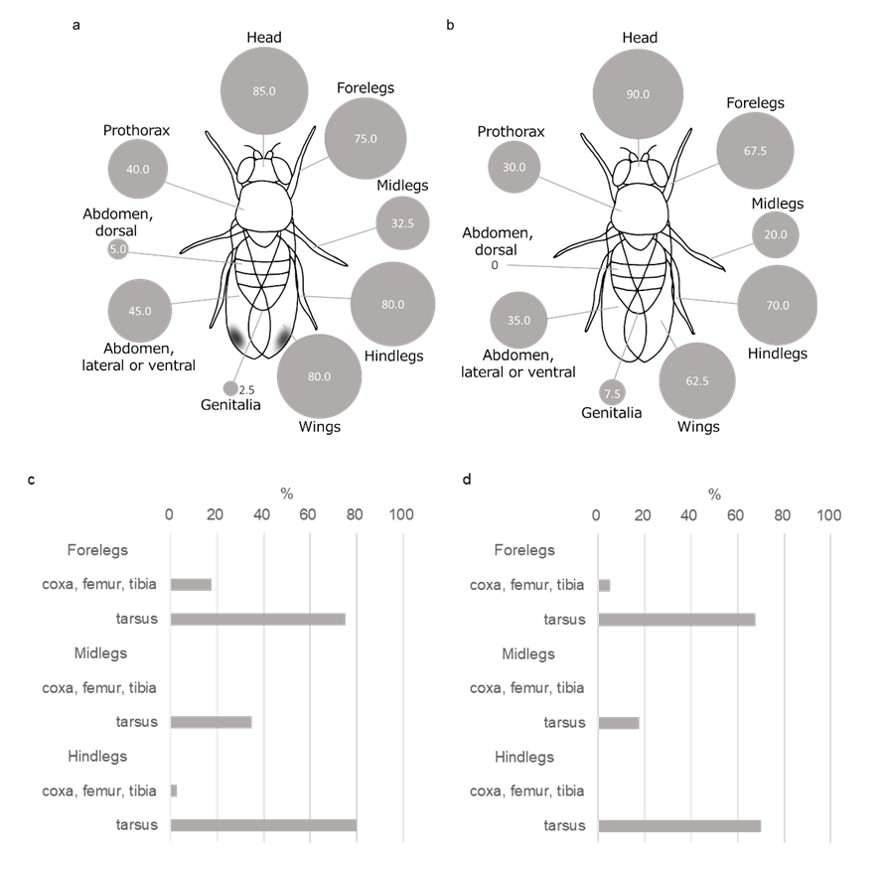

Supplement: Supplemental Material [file KFLY_A_2234265_SM3497.zip › Yamazaki et al FigS6.jpg]

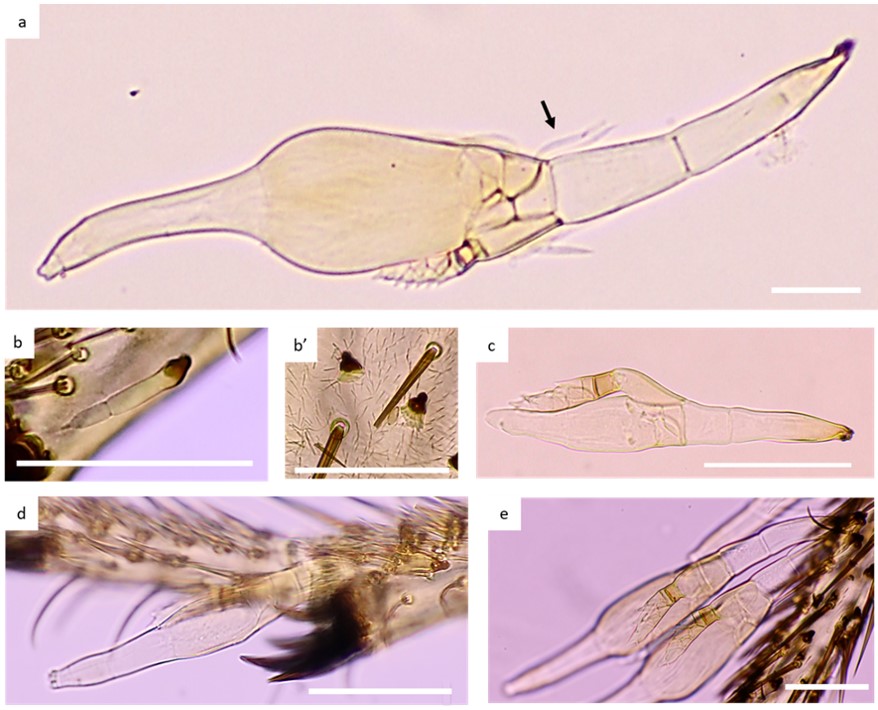

Supplement: Supplemental Material [file KFLY_A_2234265_SM3497.zip › Yamazaki et al FigS7.jpg]

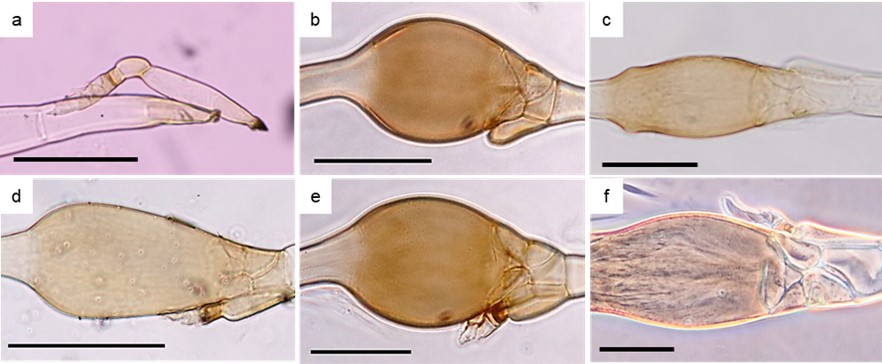

Supplement: Supplemental Material [file KFLY_A_2234265_SM3497.zip › Yamazaki et al FigS8.jpg]
